# Intraocular lens exchange through a 3.2-mm corneal incision for opacified intraocular lenses

**DOI:** 10.4103/0301-4738.73713

**Published:** 2011

**Authors:** Anil Kubaloglu, Esin Sogutlu Sari, Arif Koytak, Yasin Cinar, Kazim Erol, Yusuf Ozertürk

**Affiliations:** Kartal Dr. Lütfi Kirdar Training and Research Hospital, 2^nd^ Eye Clinic, Cevizli, Istanbul, Turkey

**Keywords:** Intraocular lens opacification, small corneal incision, intraocular lens exchange, postsurgical outcomes

## Abstract

**Aim:**

The aim was to evaluate visual and refractive results and complications of intraocular lens (IOL) exchange through a 3.2 mm corneal incision for opacified IOLs.

**Materials and Methods:**

This retrospective study comprised 33 eyes of 32 patients with IOL opacification requiring an IOL exchange between July 2003 and March 2007. Exchange surgery was performed through a 3.2-mm temporal clear corneal incision followed by implantation of a new foldable hydrophobic IOL. Uncorrected visual acuity (UCVA), best spectacle-corrected visual acuity (BSCVA), topographical astigmatism, and refractive cylinder were evaluated. Surgically induced astigmatism (SIA) was calculated and complications were recorded.

**Results:**

Opacification was observed in 25 eyes (76%) with Aqua-Sense, 3 eyes (9%) with Hydroview, 3 eyes (9%) with MemoryLens IOLs, and 2 eyes (6%) with DgR. The mean follow-up period was 36.54 months. An uneventful IOL exchange was achieved in 18 eyes (55%). Zonular dehiscence occurred in 9 eyes (27%), and posterior capsule tear developed in 4 eyes (12%). The mean preoperative BSCVA (mean ± standard deviation, decimal scale) was 0.13 ± 0.08 (mean: 20/150, range 20/2000 to 20/60) and improved to 0.63 ± 0.18 (mean: 20/32, range 20/60 to 20/20, *P* < 0.001). The mean SIA was 0.70 D. Seven eyes (21%) had 0.5 D or lower SIA.

**Conclusion:**

IOL exchange is a technically challenging procedure with potential risks of reversing the advantages of a prior small-incision cataract surgery. The use of a small corneal incision for IOL exchange could preserve the advantages of modern phacoemulsification surgery with acceptable SIA related to the procedure.

Modern phacoemulsification surgery provides less corneal distortion, less postoperative inflammation, and lower postoperative astigmatism, allowing patients to return to their daily lives within a shorter time.[1] The implantation of a foldable intraocular lens (IOL) is an essential part of modern phacoemulsification surgery. Most surgeons prefer foldable IOLs because they can be implanted through a small corneal incision and consequently may reduce surgically induced astigmatism (SIA).[2]

The opacification of an IOL is a potential complication of IOL implantation surgery. In the past decade, studies have reported the formation of calcium deposits on IOLs in intraoperative,[3] immediate postoperative,[4] or late postoperative periods.[5] IOL exchange surgery involving the explantation of the opacified IOL and implantation of a new one is sometimes the only solution for improving visual function and reducing complaints of glare and halo.[6–9]

In recent studies, several surgical techniques were used to remove the opacified IOLs, but posterior capsule ruptures and zonular dehiscence occurred in 4.3–50% of eyes during surgery because of adhesions between the capsule and the optic and the haptics of opacified IOLs.[7–11] Therefore, IOL exchange is a technically challenging procedure that may reverse the advantages of prior small-incision cataract surgery in cases of unexpected complications.

In the present study, patients with visual complaints related to IOL opacification were treated with a technique involving the explantation of the opacified IOL and reimplantation of a new one through the same incision used for the original phacoemulsification. Visual and refractive results, complications, and SIA were evaluated.

## Materials and Methods

A total of 3320 eyes of 2510 patients who had phacoemulsification surgery between July 2003 and March 2007 were retrospectively reviewed. Patients with IOL opacification that required IOL exchange surgery were included in the study. Informed consent was obtained from all patients in accordance with the Declaration of Helsinki, and the Institutional Review Board of Kartal Dr Lütfi Kırdar Training and Research Hospital, Istanbul, Turkey approved the study. Diagnosis of IOL opacification was made with biomicroscopic examination in a fully dilated pupil. A complete medical history of ophthalmic and systemic diseases was obtained from each patient. A thorough ophthalmic examination was performed preoperatively and postoperatively, including uncorrected visual acuity (UCVA), best spectacle-corrected visual acuity (BSCVA), refractive cylinder, keratometric readings using the Orbscan II (Bausch & Lomb, Rochester, NY, USA), and the presence of posterior capsule opacification (PCO). Intraocular pressure (IOP) was measured with applanation tonometry. Intraoperative and postoperative complications were also recorded. SIA was calculated by vector analysis[12] 1 month after the operation and at the last examination. All statistical analyses were made with the SPSS for Windows software (version 11.5, SPSS Inc., Chicago, IL, USA). Preoperative and postoperative UCVA, BSCVA, topographical astigmatism, and refractive cylinder were compared with the paired samples *t*-test when data were normally distributed or the Wilcoxon signed ranks test when data were not distributed normally. A *P*-value of 0.05 or less was accepted as statistically significant.

IOL exchange was performed under local subtenon anesthesia by the same surgeon (AKu). First, two side-ports were made with a 20-gauge knife. Viscosurgical devices (Viscoat, Alcon Laboratories, Fort Worth, TX, USA) were applied in the anterior chamber. The opacified IOL was separated from the capsule and taken to the anterior chamber by viscodissection. A temporal clear corneal incision at the same location of the original phacoemulsification incision was made with a 3.2-mm slit knife. The IOL was divided into two through its optic and each piece was removed through the corneal incision using forceps. When the IOL was adherent to the capsular rim, one or both haptics were cut as peripherally as possible in order to prevent zonular dehiscence. Anterior vitrectomy was performed when vitreous loss was determined. In cases of zonular dehiscence, a capsular tension ring (Morcher GmbH, Stuttgart, Germany) was implanted to provide regular capsular tension. New foldable hydrophobic IOLs (Sensar, Abbott Medical Optics Inc., Santa Ana, CA, USA) were implanted into the capsular bag when capsular integrity was maintained. These IOLs were implanted into the ciliary sulcus when capsular adherence, capsule contraction, or posterior capsular tear was present. However, they were fixated to the sclera from one haptic in cases of insufficient capsular integrity. When the opacified IOL was explanted along with the lens capsule, a new foldable hydrophobic IOL was fixated to the sclera from both haptics. After surgery, the patients received ciprofloxacin 0.3% (Ciloxan, Alcon Laboratories, Inc.) and prednisolone acetate 1% (PRED FORTE; Allergan, Inc., Irvine, CA, USA) eye drops four times per day for 1 month. Prednisolone acetate 1% eye drops were tapered off over 2 months in routine. The dosage of prednisolone acetate 1% was increased up to 12 times a day and topical cyclopentolate 1% (Cyclopentolate Thilo; Dr Thilo & Co, Gmbh, Freiburg, Germany) three times a day was ordered in eyes with dense fibrinoid reaction. Topical ketorolak trometamin 0.5% (ACULAR; Allergan, Inc.) four times a day and oral indomethacine 25 mg (Endol; Deva Lab., Tekirdag, Turkey) twice a day were added to the regime when cystoid macular edema was diagnosed with optical coherence tomography.

## Results

Thirty-three (1%) eyes of 32 patients out of 3320 eyes had visually significant IOL opacification and had IOL exchange surgery. The presence of PCO was seen in 346 eyes (10%). IOL exchange was performed in 11 male (34%) and 21 female (66%) patients. The main indications for IOL exchange were poor visual acuity, glare and/or halos and compromised view of the posterior segment with diabetic retinopathy. However, 37% of patients (12 eyes) complained of decreased vision, 15% (5 eyes) complained of glare and/or halos, and 39% (13 eyes) complained of both. IOL exchange was indicated in three patients (9%) in order to have a better view of fundus for the management of diabetic retinopathy. At the time of surgery, the mean age was 66.54 ± 11.03 years (mean ± SD [standard deviation], range 55–89 years). Opacification was diagnosed during follow-up visits after uncomplicated phacoemulsification surgery for senile cataracts. The mean time interval between the cataract surgery and IOL exchange was 27.69 ± 11.18 months (range 8–56 months). The mean follow-up period was 36.54 ± 16.97 months (range 24–67 months). All patients completed the second year examination. Two eyes (6%) had Nd:YAG laser capsulotomy due to poor vision prior to the IOL exchange surgery.

Opacification was observed in 25 eyes (76%) with Aqua-Sense (Ophthalmic Innovation International, Ontario, CA, USA), 3 eyes (9%) with Hydroview (Bausch & Lomb), 3 eyes (9%) with MemoryLens (IOL Tech. Lab., Cedex, France) and 2 eyes (6%) with DgR (Medical Development Research, Inc., USA). All opacified IOLs were made up of a hydrophilic acrylic material. Concurrent intraocular pathologies were age-related macular degeneration in two eyes (6%), pseudoexfoliation syndrome in three eyes (9%), and primary open-angle glaucoma in one eye (3%). Three of the patients (9%) had diabetes mellitus as a systemic pathology, one patient (3%) had chronic renal failure, and one patient (3%) had hypertension.

Visual acuity was measured with Snellen charts. The mean UCVA was 0.017 ± 0.020 preoperatively, 0.26 ± 0.16 at 1 month, and 0.44 ± 0.14 at last examination (*P* < 0.001). The mean BSCVA was 0.13 ± 0.08 preoperatively, 0.62 ± 0.19 at 1 month, and 0.63 ± 0.18 at last examination (*P* < 0.001). The mean topographical astigmatism was 1.05 ± 0.48 diopters (D) before the IOL exchange, 1.09 ± 0.45 D at the first postoperative month and 1.09 ± 0.48 D at the last examination (*P* = 0.57 and *P* = 0.50, respectively). The mean manifest cylinder was –0.62 ± 0.40 D before the IOL exchange, 0.74 ± 0.26 D at the first postoperative month, and –0.78 ± 0.33 D at the last examination (*P* = 0.06 and *P* = 0.07, respectively). The mean SIA was 0.68 ± 0.21 D after 1 month and 0.70 ± 0.24 D at the last examination. Table 1 shows the mean UCVA, BSCVA, topographical astigmatism, and refractive cylinder before and after surgery.

**Table 1:** UCVA, BSCVA, topographical astigmatism, and refractive cylinder before surgery, after 1 month, and on last examination (mean = 36.5 months)

Mean	Before surgery	First month	Last examination	*P*-value
UCVA (Snellen)	0.017 ± 0.020	0.26 ± 0.16	0.44 ± 0.14	<0.001
BSCVA (Snellen)	0.13 ± 0.08	0.62 ± 0.19	0.63 ± 0.18	<0.001
Topographical astigmatism (D)	1.05 ± 0.48	1.09 ± 0.45	1.09 ± 0.48	≥0.05
Manifest cylinder (D)	–0.62 ± 0.40	–0.74 ± 0.26	–0.78 ± 0.33	≥0.05

D: diopter; UCVA: Uncorrected visual acuity; BSCVA: Best spectaclecorrected visual acuity.

A complete separation of the opacified IOL from the capsular bag without complication was achieved in 18 eyes (55%). However, partial zonular dehiscence was seen in eight eyes (24%), total zonular dehiscence in one eye (3%), and posterior capsule rupture developed in four eyes (12%) during the surgery. In eyes with total zonular dehiscence, the opacified IOL was removed along with the lens capsule. Adhesions between the IOL haptics and the capsular bag were observed in two eyes (6%). Both IOL haptics were cut in these eyes to avoid stress to the zonules. No complications related to retained haptics were observed.

Anterior vitrectomy was performed in five eyes (15%). Anterior vitrectomy was applied for the management of vitreous prolapse due to partial zonular loss in one eye, total loss of zonules in one eye, and a ruptured posterior capsule in three eyes. Seven eyes with partial zonular dehiscence were managed successfully with capsular tension rings and secondary foldable hydrophobic IOLs were implanted within the bag. In one eye with partial zonular dehiscence, the new IOL was placed in the ciliary sulcus because of insufficient capsular support, and transscleral fixation was performed from one haptic. All eyes with a ruptured posterior capsule were managed with sulcus IOL implantation. Secondary foldable hydrophobic IOLs were implanted within the bag in 25 eyes (76%) and in the ciliary sulcus in 7 eyes (21%). Scleral fixation from both haptics was performed in one eye (3%) with total zonulolysis. In this study, all opacified IOLs were exchanged with a new foldable hydrophobic IOL. Operative complications and new IOL positions are summarized in Table 2.

**Table 2:** Operative complications and new IOL position

Complications	n/Percentage
Partial zonulolysis	8/24.2
Total zonulolysis	1/3.0
Posterior capsule rupture	4/12.1
Vitreous loss	5/15.1
Haptics left	2/6.0
Implantation of capsular tension ring	7/21.2
New IOL position	
Bag	25/75.7
Sulcus	7/21.2
Scleral fixation by	
One haptic	1/3
Both haptics	1/3

IOL: Intraocular lens

Postoperatively, a transient increase in IOP occurred in one eye (3%) and cystoid macular edema occurred in one eye (3%). At 38 months after IOL exchange surgery, IOL dislocation occurred in 1 eye (3%) and *ab externo* transscleral fixation of one haptic was performed. Glare and halos were not observed in any eye.

## Discussion

The delayed opacification of foldable IOLs is a potential complication of IOL implantation following phacoemulsification surgery. Opacification is related to local agents (viscoelastic devices[13] and mitomycin C)[14] and systemic conditions (diabetes mellitus,[15–17] hypertension,[16] ischemic heart disease,[16] and uveitis)[17] among other factors. Histopathologic studies using light microscopy, scanning electron microscopy, and X-ray spectroscopy showed that opacification was caused by granular deposits composed of calcium phosphate hydroxide under the surface of the IOL[18–20] in some cases. However, Dorey *et al*. reported that opacification is linked to the migration of silicone from the SureFold packaging followed by the gravitation of calcium ions to the Hydroview surface.[17] Although postoperative opacification has been reported in several studies,[13–15,17–20] there have been relatively few reports regarding the surgical outcomes of IOL exchange surgery due to IOL opacification. In this paper, we report visual and refractive results as well as complications and the astigmatic effect of opacified IOL explantation followed by a new foldable IOL implantation through a small corneal incision.

There have been a number of clinical reports regarding the phenomenon of late postoperative opacification of hydrophilic acrylic IOLs, including Hydroview,[21,22] the SC60B-OUV,[23] and Aqua-Sense.[8] In our study, opacification was assessed in 33 eyes; most of them were Aqua-Sense lenses (76%).

The intraoperative complication rates in our study were comparable to those of previous reports. Yu *et al*. reported 2 (13%) posterior capsule ruptures and 3 (20%) zonular dehiscence in a series of 15 explantations of opacified Hydroview IOLs.[7] Also, Dagres *et al*. encountered higher rate complications than other studies; they reported 11 (50%) zonular dehiscence and 2 (9%) posterior capsule ruptures in 22 explanted opacified Aqua-Sense IOLs.[8] In our study, zonular dehiscence was seen in 9 eyes (27%) and posterior capsule rupture occurred in 4 eyes (12%) in a series of 33 opacified IOLs. Another study[24] reported a 23% rate of vitreous loss necessitating anterior vitrectomy whereas in our series anterior vitrectomy was performed in five eyes (15%).

Recently, studies have reported that when a posterior capsule tear occurs during phacoemulsification surgery, a foldable IOL can be placed in the sulcus if zonular support is sufficient.[25,26] In this study, we managed the posterior capsule tear successfully with the implantation of foldable IOLs placed into the ciliary sulcus. However, some investigators managed IOL dislocation or aphakia after phacoemulsification with the transscleral fixation of foldable IOLs from haptics.[27,28] In the current study, capsular integrity was not achieved in one eye, and then transscleral fixation was performed from one haptic. Postoperative IOL dislocation developed in one eye (3%) and *ab externo* transscleral fixation was performed.

Recently Lee *et al*.[11] reported a new technique in which the intraocular opacified lens was exchanged with removal of the optic only to avoid zonular dehiscence. One or both haptics were left in the capsule and new foldable IOLs were implanted into the bag (39%) or into the ciliary sulcus (57%) and zonular dehiscence developed in 4% of eyes. They observed no complication associated with haptic dislocation with the positioning of a newly implanted IOL. However, 26% of eyes reported glare due to the progression of capsule opacity, which covered the remnant haptics and intermittent recurrent anterior uveitis developed in 4% of eyes. In our study zonular dehiscence developed in nine eyes (27%) and was successfully managed with capsular tension rings in seven of them, and a new foldable IOL was implanted in the capsular bag. However, adhesions between the IOL haptics and the capsular bag were seen in two eyes (6%) in which both haptics were cut and left in place to avoid stress to the zonules and a new foldable IOL was implanted into the ciliary sulcus.

Clear corneal, limbal, or scleral tunnel incisions are applied for IOL exchange in different studies.[29,30] The main advantage of scleral tunnel incision is that lesser astigmatism is induced when the incision is extended. In our study, we have used the same incision site of the original phacoemulsification for IOL exchange and we did not face any intra- or postoperative complication related to the incision site. Similarly, several studies report that IOL implantations within the capsular bag and within the ciliary sulcus were found equally effective and safe in a 5-year follow-up period.[29–31]

The strong adhesions between the capsule and the opacified IOL makes the implantation of a new foldable IOL through small corneal incision difficult following an IOL exchange surgery.[8] Because of the zonular dehiscence or posterior capsule rupture, sulcus IOL implantation or scleral fixation was performed with rigid poly(methyl methacrylate) IOLs.[7,8] Dagres *et al*.[8] performed IOL exchange with a 5.5-mm corneal incision that was closed with a 10/0 nylon material. Vector analysis demonstrated that corneal tunnel incisions induced astigmatism depending on incision size. Kohnen *et al*.[32] compared the induced astigmatism after temporal clear corneal incisions of different sizes. After 6 months, SIA was found to be 0.56 D after foldable IOL implantation through a 4-mm incision following phacoemulsification surgery. In this study, all opacified IOLs were exchanged with new foldable IOLs through a temporal 3.2-mm clear corneal incision. The mean SIA was 0.68 ± 0.21 D after 1 month, and there was no statistically significant difference between the preoperative and postoperative topographical astigmatism and refractive cylinder. When IOL opacification develops, the opacified IOL can be explanted through small corneal incision and a new foldable IOL can be implanted. This procedure may lead to acceptable SIA.

Published studies have demonstrated various visual outcomes. Some[10,11] reported significant visual improvement, whereas other[8] reported no significant improvement in visual acuity. In our study, the mean best corrected visual acuity was 0.13 ± 0.08 preoperatively, and 0.63 ± 0.18 at the last examination (*P* < 0.001). This statistically significant improvement in the visual acuity may be explained by faster visual rehabilitation and less corneal astigmatism after the implantation of the foldable IOLs through small corneal incision.

Consequently, the implantation of new foldable IOLs in eyes developing a posterior capsule tear or zonular dehiscence during IOL exchange surgery for opacified lenses provides the advantage of a small corneal incision, working in a closed system, decreasing surgically induced astigmatism, faster wound healing, and early visual rehabilitation.
